# Multi-objective node placement optimization in multiplex 6G wireless networks using quantum-inspired evolutionary algorithms

**DOI:** 10.1038/s41598-026-56463-z

**Published:** 2026-06-04

**Authors:** Dhananjai VS, Sathi Sailesh Reddy, K Abhimanyu Kumar Patro, Soumya Ranjan Das

**Affiliations:** Manipal Institute of Technology, Manipal Academy of Higher Education, Manipal, India

**Keywords:** 6G wireless networks, Multiplex networks, Node placement optimization, Quantum-inspired evolutionary learning, Multi-objective optimization, Engineering, Mathematics and computing

## Abstract

The planning of future sixth-generation (6G) wireless networks needs efficient node placement algorithms that can handle heterogeneous network deployment, high connectivity density, and competing performance requirements. Current node placement algorithms mostly use single-layer graph models and traditional optimization methods, which are inadequate to model the interdependent relationship of future 6G networks. In this paper, we present a quantum-inspired evolutionary learning-based multi-objective optimization framework for node placement in multiplex 6G wireless networks. The network is represented as a multiplex graph to capture the interactions between the capacity, latency, and interference layers. The solutions are probabilistically represented using quantum-inspired representations to facilitate global exploration and prevent premature convergence in the high-dimensional search space. A composite fitness expression is designed to address the optimization of network capacity contribution, incentive-aware participation, and multi-layer node centrality simultaneously. Extensive simulations validate that the proposed framework achieves a balanced fitness score of 3.0355 and a *75.6%* node cooperation rate. Compared to existing methods, the QIEA framework improves overall deployment fitness by *61%* over random selection and *12%* over greedy degree based placement. Furthermore, it converges *23%* faster than standard Genetic Algorithms (GA), demonstrating superior efficiency and scalability for largescale 6G network optimization.

## Introduction

The upcoming Sixth-Generation (6G) wireless communication technology will re-engineer the concept and operation of telecom networks. With peak data rates of terabits per second and end-to-end latency of microseconds, 6G will enable immersive metaverse, holographic, ultra-reliable autonomous, and mission-critical cyber-physical services^1^. To this end, a paradigm shift in network design from homogeneous networks to highly heterogeneous, ultra-dense, and intelligence-driven networks is required^2^.

In contrast to previous generations, 6G will integrate the following: terrestrial small cells, Low Earth Orbit (LEO) satellites, Unmanned Aerial Vehicles (UAVs), and edge computing^2^. Node and Virtualized Network Functions (VNF) location are very important for performance; suboptimal location may increase end-to-end delay, inter-cell interference, and power consumption^3,4^. The problem is a restricted combinatorial optimization problem, which is NP-hard in large 6G networks, so exhaustive search and traditional algorithms are not feasible even for small networks^5,6^.

Recent studies on 6G planning have indicated that network performance is not a function of one variable but rather more than one variable. The performance of the deployment node is a function of multiple layers such as capacity, delay, and interference, which are dynamic in nature and cannot be modeled using graphs that are single-layered^7,8^. Multiplex network modeling is an area that has great potential in modeling next-generation wireless networks^9^.

Node location in 6G is more than architecture and modeling; it has several conflicting objectives. Although maximizing network capacity is a priority, it has to be done in a way that reduces interference, latency, and robustness of deployment^10,11^. The decentralized and possibly multi-owner nature of 6G also introduces strategic considerations. Incentive compatibility is necessary to encourage autonomous nodes to cooperate towards achieving the objectives. Although important, incentive-aware node location has received very little attention in the literature of wireless optimization^12–14^.

From an algorithmic perspective, traditional metaheuristics such as Genetic Algorithms (GA) and Particle Swarm Optimization (PSO) are popular for node location and network function placement^15–18^. However, they are challenged by the high-dimensional and multi-objective nature of 6G networks. GAs and PSO are prone to premature convergence because of the loss of diversity, resulting in suboptimal solutions, and they converge slowly, incurring high computational complexity–planning can take days or weeks, which is not suitable for dynamic planning and real-time reconfiguration in 6G^19^. Although ML-based optimization techniques have been investigated, their poor generalizability in different topologies and scenarios often demands retraining^20–24^.

Motivated by the above-mentioned issues, this paper proposes an approach based on the use of Quantum-Inspired Evolutionary Algorithms (QIEAs) for multi-objective node placement optimization in multiplex 6G wireless networks. One of the key limitations associated with current research into 6G node placement and wireless optimization is the need to balance modeling accuracy and computational tractability. On one hand, most of the existing optimization frameworks make use of oversimplified single-layer network models to ensure that the problem remains computationally feasible for evolutionary algorithm implementation; on the other hand, most frameworks using realistic multilayer communication models resort to heuristics that do not allow efficient optimization because of the combinatorial explosion of the search space. Therefore, the current frameworks are unable to concurrently achieve scalability, multilayer dependency modeling, and multi-objective optimization for deployment in ultra-dense 6G networks.

The first novel aspect of the proposed framework is the synergy between multiplex network modeling and probabilistic optimization based on QIEA. While the conventional frameworks consider network topology as a static optimization space, the proposed framework exploits the properties of multilayer topologies for the sake of achieving optimization scalability and efficiency. The choice of QIEA as an optimizer is based on the inability of other meta-heuristics, such as Genetic Algorithms (GA) and Particle Swarm Optimization (PSO), to perform efficient and scalable optimization in ultra-dense and high-dimensional search spaces inherent in multiplex 6G network topologies. In particular, in the case of node placement problems with hundreds of potential nodes, the conventional evolutionary optimization methods face an issue of quick population diversity degradation and premature convergence to local optima^15,16,18^. These limitations assume greater significance in multiplex 6G communication systems, which are subject to coupled latency, interference, and connectivity limitations in multiple interdependent network layers.

To counter this problem, the suggested approach utilizes quantum-based optimization techniques, especially quantum superposition and probability-based qubit encoding technique, in the evolutionary algorithm^25–27^. In contrast to the standard binary encoding technique, the qubit is encoded as

1 $$\begin{aligned} |\psi _i\rangle = \alpha _i |0\rangle + \beta _i |1\rangle \end{aligned}$$

where $|\alpha _i|^2 + |\beta _i|^2 = 1$. The above representation allows one to probabilistically encode many deployment states into one quantum chromosome, leading to improved exploration ability of the optimization algorithm.

The probabilistic encoding of quantum bits ensures that the population remains highly diverse over time, preventing premature convergence to suboptimal solutions. Additionally, evolving probability amplitudes $(\alpha _i,\beta _i)$ rather than fixed binary strings facilitates robust and efficient convergence towards optimal deployment states, which is critical to meet adaptive near real-time reconfiguration demands of future intelligent 6G networks.

Furthermore, the suggested approach incorporates an advanced multiplex-aware candidate reduction approach which uses multilayer centrality assessment and structure-based filtering within both latency and interference layers for drastically reducing the search space before applying evolutionary computation techniques. Through limiting optimization to structurally significant candidate nodes, the approach increases its computational feasibility and scalability in highly dense deployment scenarios.

Through the consideration of the communication network infrastructure as a multiplex graph, the suggested approach can represent the interdependencies between the layers of latency, interference, and connectivity. The performance of node deployment schemes is assessed based on a multi-objective fitness function that maximizes the contributions of capacity provision, incentive compatibility, and multilayer node centrality^28–32^. The suggested QIEA-based probabilistic optimization approach conducts an adaptive multi-objective search over the compact multiplex solution space through qubit-based probabilistic representation and evolutionary amplitude evolution. Such an approach enhances exploration capability, convergence stability, and cooperative node selection compared to conventional binary evolutionary optimization approaches.

Hence, by integrating multiplex structural intelligence with quantum-inspired probabilistic optimization in a synergistic manner, the above framework provides solutions to the issues related to scalability, multi-layer dependency analysis, implementation feasibility, and adaptive multi-objective optimization within next-generation intelligent 6G wireless network architectures without compromising on robustness and scalability in optimization.

The present study makes a contribution towards the achievement of Sustainable Development Goals (SDGs) of the United Nations through development of efficient, resilient and intelligent communication networks. Specifically, the current framework is relevant to SDG 9 (Industry, Innovation, and Infrastructure) by enhancing the scalability of optimization approaches for next-generation 6G network design. In addition, through the improvement of resource utilization and energy savings by optimizing node placement, the study further addresses SDG 7 (Affordable and Clean Energy) and SDG 11 (Sustainable Cities and Communities).

The rest of the paper is structured as follows. The section entitled “Background and Related Work” gives the basic concepts involved in multiplex 6G wireless networks, evolutionary optimization algorithms, network centrality, network function virtualization and placement, network robustness approaches, and performance measures and multi-objective optimization. The section entitled “Proposed Methodology” provides the details of the proposed QIEA framework, which includes multiplex network generation, candidate nodes selection, quantum-inspired evolutionary optimization, and multi-objective fitness evaluation methods. The section entitled “Results and Discussion” provides the results of the experiments conducted, the optimization performance, convergence behavior, and comparative analysis of the proposed approach. Finally, the section entitled “Conclusions and Future Work” concludes the main contribution of the study along with possible future research directions in adaptive and large scale deployment optimization for 6G networks.

## Background and related work

### Multiplex networks in 6G infrastructure

Multiplex networks model real-world systems in which a set of nodes interact with each other in multiple layers, each of which has its own set of edge weights. In the context of 6G wireless networks, these layers are representative of operational constraints such as latency, interference, and capacity. These layers are necessarily interdependent, where the performance of one layer affects the performance of the other layers. This makes it difficult to draw conclusions about the health of the network and the criticality of nodes based on individual layers^7,9^.

It has been observed in previous research that the projection of multi-layer networks onto single-layer spaces affects both topological structure and dynamics. Specifically, Interdonato et al.^33^ proved that single-layer representations are ineffective in maintaining the importance and interaction of nodes in heterogeneous wireless networks. On this basis, Battiston et al.^8^ further generalized multiplex models to complex wireless networks and proved that inter-layer coupling not only enhances topological accuracy but also greatly simplifies analysis compared to traditional aggregated network models. The above results make multiplex network modeling a basic need in 6G infrastructure design, where a node chosen for high capacity in one layer should not become a latency bottleneck or interference hotspot in another^9^.

### Evolutionary algorithms and quantum-inspired optimization

Evolutionary and population-based optimization algorithms such as GA, PSO, and Differential Evolution (DE) are popular in the area of network optimization (routing, scheduling, resource allocation)^15–18^. However, their performance deteriorates rapidly in the high-dimensional, exponentially large solution spaces of modern 6G networks, particularly when the dimensionality of optimization exceeds a few hundred. The use of fixed binary/real-valued codings and local search operators tends to trap these algorithms in suboptimal local optima^19,34,35^.

To overcome these drawbacks, Quantum-Inspired Evolutionary Algorithms (QIEAs) combine quantum ideas, particularly superposition, with traditional evolutionary algorithms. By leveraging the probability amplitudes of qubits to represent solutions, QIEAs allow for the exploration of multiple areas within the solution space concurrently. Han showed that the solution representation scheme improves convergence speed over traditional genetic algorithms^25,27^. Zhang et al.^28^ and Narayanan and Moore^26^ have also verified the efficacy of QIEAs for difficult combinatorial problems. The results show that a proper integration of evolutionary search with quantum-inspired solution representation makes QIEAs applicable to large-scale network topology optimization problems.

More recently, quantum-inspired optimization methods have started to gain interest in the area of wireless networking. For example, Lin *et al.* have used a quantum-inspired Coherent Ising Machine to solve a multi-objective routing optimization problem in wireless multi-hop networks, showing the benefits of improved solution quality for complex combinatorial search problems^36^. Taghavi and Vahidi have proposed a quantum-inspired multi-agent reinforcement learning approach for UAV-assisted 6G network deployment, with a focus on exploration-exploitation trade-offs in dynamic coverage optimization problems^37^. In a similar vein, Narottama *et al.* have used quantum-inspired evolutionary algorithms for NOMA user pairing, showing the benefits of probabilistic quantum-inspired representations for wireless communication optimization problems^38^.

Although these studies verify the effectiveness of quantum-inspired approaches for optimizing wireless networks, they mainly focus on single-layer abstractions of wireless networks or certain deployment tasks, like routing optimization, coverage, and user pairing. They are not designed to model the dependencies of multiplex networks explicitly or to handle incentive-aware multi-objective node placement in large-scale 6G wireless networks. In contrast, the proposed approach combines quantum-inspired evolutionary learning with multiplex network modeling to optimize the interlinked latency, interference, and capacity properties of wireless networks, along with incentive compatibility for cooperative node placement in 6G wireless networks.

### Network centrality measures for node selection

The network centrality measures are very important tools for the detection of structurally significant nodes in complex networks. Specifically, the betweenness centrality measures the extent to which a particular node is on the shortest paths between other pairs of nodes, thus capturing the node’s function of information control. Freeman grouped centrality measures into three types: degree centrality, closeness centrality, and betweenness centrality^10,11^.

With regard to network function placement, nodes with high betweenness centrality values tend to represent traffic aggregation and relay points, which require greater capacity. Nevertheless, single-layer centrality analysis is inadequate in the multiplex 6G network setting, where a node that is highly central in the latency layer may be inefficient or even isolated in the interference layer. To overcome this drawback, multiplex centrality analysis, including multi-degree and strength centrality, assess node centrality in multiple layers concurrently^12–14^. Comparison studies have demonstrated that multiplex centrality analysis performs better than single-layer centrality analysis by 23–31% in pinpointing truly critical nodes for robust infrastructure placement, establishing their significance in 6G network design.

### Network Function Virtualization and Placement Strategies

Contemporary telecommunication systems are progressively dependent on frameworks that utilise virtualisation, separating network functions from specific hardware and implementing them as virtual entities on common physical infrastructure^5^. This framework presents a significant placement challenge that entails choosing suitable nodes for the instantiation of VNFs, all while ensuring that quality-of-service (QoS) criteria like latency and bandwidth are met^29,39^.

The optimal VNF placement is provided by Integer Linear Programming (ILP), but ILP becomes too complex for large networks, so we usually use heuristic and approximation algorithms^30^. Recently, researchers have begun to use machine learning, particularly deep reinforcement learning, for VNF placement. However, generalisation is difficult when the network topology varies, so we must use other optimisation methods^24^.

### Topology robustness and evolutionary node placement optimization

Recent studies have increasingly focused on improving topology robustness and resilient node placement strategies for large-scale IoT and Industrial Internet of Things (IIoT) environments through intelligent evolutionary and learning-based optimization techniques. In highly dynamic communication infrastructures, maintaining robust connectivity under varying network conditions, failures, and cyberattacks has become a critical research challenge. To address this issue, Chen *et al.* proposed an adaptive Bayesian learning framework for fast robustness enhancement in dynamic IIoT topologies, enabling rapid global topology optimization under continuously evolving network conditions through adaptive continuous coding strategies^40^. Similarly, Qiu *et al.* introduced a self-adaptive robustness optimization method with an evolutionary multi-agent system (ROMEM), where collaborative co-evolutionary learning mechanisms significantly improved global exploration capability and convergence efficiency for topology adaptation problems^41^.

Concurrently, structure optimization techniques based on motifs have been proposed as a means of enhancing the resilience of networks. The LEGO-Motif approach employs evolutionary motif-based topological design to enhance the resilience of IoT systems against disruptions and cyber attacks via sequential motif addition informed by evolutionary learning^42^. Similarly, DACMotif adopts a divide-and-conquer evolutionary optimization method to improve robustness in large-scale IoT networks, where the computational burden is significantly reduced with a view to avoiding premature convergence^43^. Although these methods demonstrate strong robustness and scalability characteristics for single-layer IoT and IIoT environments, they are not specifically designed for multiplex 6G wireless infrastructures involving coupled latency, interference, and capacity dependencies across multiple interconnected network layers. In contrast, the proposed Quantum-Inspired Evolutionary Algorithm (QIEA) framework extends evolutionary robustness optimization into multiplex network environments through multi-objective node placement optimization considering interdependent multilayer network characteristics.

### Energy-efficient hybrid optimization in wireless sensor networks

Recent research trends in wireless sensor networks (WSNs) and wireless communication systems have increasingly focused on energy-efficient hybrid optimization techniques to improve network lifetime, routing efficiency, clustering stability, and communication reliability. Hybrid bioinspired optimization frameworks have been widely explored to address the challenges associated with energy-aware node selection, cluster-head optimization, hotspot mitigation, and secure wireless communication in resource-constrained network environments.

Lonkar and Karmore proposed the BCEWN framework, a hybrid bioinspired clustering model for energy-aware wireless network deployment that combines intelligent clustering mechanisms with energy-efficient node management strategies to improve network lifetime and communication efficiency^44^. Similarly, recent studies on hybrid cluster-head selection strategies have demonstrated that combining multiple optimization techniques can significantly improve energy balancing, communication overhead reduction, and routing stability in large-scale WSN environments^45^. In addition, statistical evaluation of power-aware routing protocols has shown that adaptive routing optimization plays an important role in reducing communication energy consumption and improving network sustainability under dynamic traffic conditions^46^.

Recent advancements have also explored blockchain-assisted and bioinspired security optimization methods for wireless communication networks. The EBBSMWN framework integrates blockchain mechanisms with bioinspired optimization strategies to improve energy-aware security and resilient communication in wireless network infrastructures^47^. Moreover, recent literature reviews of hybrid clustering and routing techniques have pointed out that hotspot creation, unequal energy dissipation, and scaling problems are still the primary unresolved issues in large-scale WSN implementations, even though there exist a number of hybrid optimization techniques^48^. These reviews stress the increasing demand for scalable and adaptive optimization schemes that can address all three aspects at once.

Although these methods provide effective solutions for energy-efficient communication, clustering optimization, and routing management in traditional WSN and wireless network environments, most existing approaches primarily focus on single-layer architectures and localized optimization objectives. They do not explicitly address the interdependent multilayer characteristics associated with multiplex 6G infrastructures involving coupled latency, interference, connectivity, and capacity constraints. In contrast, the proposed Quantum-Inspired Evolutionary Algorithm (QIEA)-based framework performs multi-objective node placement optimization within multiplex 6G wireless environments by jointly considering multilayer structural dependencies and adaptive evolutionary optimization mechanisms.

### Performance metrics and multi-objective optimization

The optimization of 6G infrastructure requires the simultaneous evaluation of multiple performance criteria. From the existing literature, it is clear that the deployment of 6G infrastructure needs to be done while considering connectivity along with quality-of-service criteria such as latency and interference, apart from economic criteria such as incentive compatibility^49,50^. These criteria are conflicting in nature since improvement in one criterion results in deterioration in another.

Several multi-objective optimization approaches, such as Pareto front approximation algorithms, have been widely used to deal with these issues^31^. In real-world deployment scenarios, weighted sum formulations are generally used to transform these conflicting objectives into a single objective function that reflects real-world priorities. The use of suitable weights for capacity contribution, incentive alignment, and network centrality makes it possible to achieve a balanced optimization that meets the operational needs of future 6G networks.

While the suggested framework uses a static formulation of the weighted sum approach for assessing multiple objectives fitness, real-world 6G wireless networks can be subject to changing priorities under varying traffic loads, latency demands, interferences, power limitations, and cooperation among distributed nodes. The weights used in the current research have been chosen to ensure computational efficiency and interpretability as well as to serve as a baseline for balanced optimization of multiple objectives considered.

However, static scalarization can be an obstacle to adaptability in face of constantly changing conditions of deployment. The future research direction that should be considered in relation to the developed optimization framework is related to adaptive Pareto-front based multi-objective optimization techniques that will be able to generate non-dominated solution sets for dynamic decision making processes. The application of such strategies would enable network providers to freely choose their deployment strategies based on their operational needs without having to completely redefine their optimization strategies.

## Proposed methodology

In this section, the proposed methodology based on quantum-inspired evolutionary approach to solve the multi-objective node placement problem in multiplex 6G wireless networks is described. The methodology includes concepts from multiplex network theory, centrality-based candidate reduction, and quantum-inspired evolutionary optimization to address scalability and robustness of the problem.

### Network topology modeling and preprocessing

Consider the network topology is represented by the set of nodes

2 $$\begin{aligned} \mathscr {V} = \{v_1, v_2, \ldots , v_N\}, \end{aligned}$$

where *N=542* stands for the number of potential candidate locations.

The topology datasets used in this study were retrieved from publicly available research repositories, where simulation-based evaluation approaches are applied, since there are no real-world 6G telecommunication infrastructure datasets with detailed multilayer features (such as latency, interference, and capacity) available to the public. This approach is also common in studies on emerging 6G infrastructure, since the large-scale operator datasets are not publicly available at the moment.

To increase practical relevancy, the generated 542-node multiplex network topology is designed to have key structural characteristics expected in future telecommunication infrastructures: sparsity, heterogeneous degree distribution, hub-dominated topology dynamics, and multilayer dependencies. The validity of these assumptions is discussed in the Results and Discussion section.

Two matrices are given:

- Latency matrix $$\textbf{L} \in \mathbb {R}^{N \times N}$$,
- Interference matrix $$\textbf{I} \in \mathbb {R}^{N \times N}$$.

In order to make different metrics comparable, both matrices are scaled using min–max normalization:

3 $$\begin{aligned} X_{\text {norm}}(i,j) = 2 \cdot \frac{X(i,j) - X_{\min }}{X_{\max } - X_{\min }}, \quad X \in \{\textbf{L}, \textbf{I}\}. \end{aligned}$$

Symmetry of the resulting matrices is checked to confirm the undirected connectivity assumption:

4 $$\begin{aligned} X_{\text {norm}}(i,j) = X_{\text {norm}}(j,i). \end{aligned}$$

The preprocessing pipeline of multiplex network topology normalization and validation is described in Algorithm 1.

**Algorithm 1:**
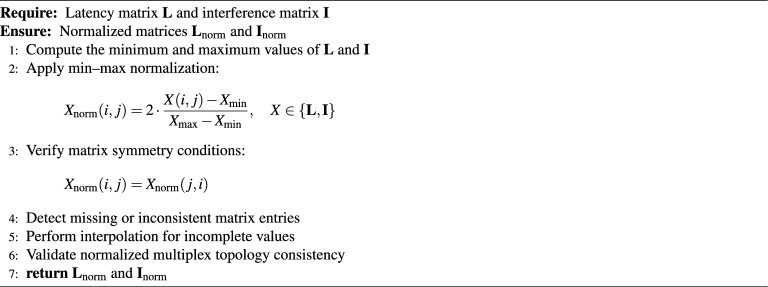
Topology Preprocessing

### Multiplex network construction

The 6G network can be considered a multiplex graph where different layers are defined over the same set of nodes. A multiplex adjacency matrix can be built as

5 $$\begin{aligned} \textbf{A}_{\text {multi}} = \textbf{A} \odot \left( \textbf{L}_{\text {norm}} + \textbf{I}_{\text {norm}} \right) , \end{aligned}$$

where $\textbf{A}$ is the binary connectivity matrix and *⊙* is the element-wise multiplication operator.

The proposed multiplex adjacency formulation provides a mathematical way of capturing the dependency between layers in future 6G networks. The binary connectivity matrix $\textbf{A}$ serves as a topological mask using the element-wise Hadamard product *(⊙ )*, which means that the optimization will be done only over possible connections. The summation of normalized latency and interference matrices gives the penalty weight for each link of the network topology.

Therefore, the larger the value of $\textbf{A}_{\text {multi}}(i,j)$ is, the more likely the physical connection has been degraded by both latency and interference. In this way, the problem of heterogeneous multi-layer networks is converted to an optimization problem using evolutionary algorithms.

The multiplex approach allows modeling several constraints on interdependent communication within one network infrastructure. Thus, the multiplex approach enhances the accuracy of node placement evaluation for future 6G environments.

The steps of the proposed multiplex adjacency construction are described in Algorithm 2.

**Algorithm 2:**
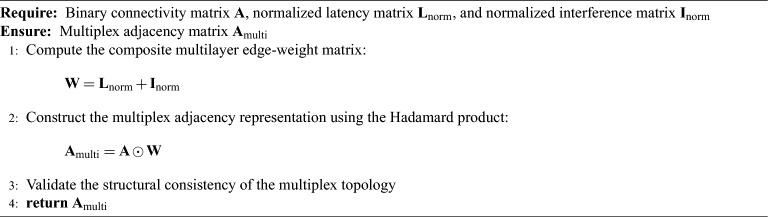
Multiplex Network Construction

### Centrality theory and candidate node reduction

The structural importance of nodes is quantified using betweenness centrality:

6 $$\begin{aligned} BC(v_i) = \sum _{s \ne v_i \ne t} \frac{\sigma _{st}(v_i)}{\sigma _{st}}, \end{aligned}$$

where $\sigma _{st}$ denotes the number of shortest paths between nodes *s* and *t*.

Node strength centrality is defined as

7 $$\begin{aligned} S(v_i) = \sum _{j} \textbf{A}_{\text {multi}}(i,j). \end{aligned}$$

To evaluate candidate suitability, local nodal metrics are derived from the global multiplex adjacency representation. The effective nodal latency, denoted as $L(v_i)$, is computed as the average normalized latency associated with all active communication links incident to node $v_i$. A node is promoted to the reduced candidate set only if it satisfies multiple structural and performance constraints simultaneously.

This multicriteria filtering mechanism ensures that isolated edge nodes with weak connectivity and nodes operating under severe latency or interference bottlenecks are mathematically pruned prior to quantum evolutionary optimization. As a result, the dimensionality of the optimization problem is significantly reduced while preserving structurally important and operationally viable candidate nodes.

A node $v_i$ is selected as a candidate if

8 $$\begin{aligned} v_i \in \mathscr {S} \iff {\left\{ \begin{array}{ll} \deg (v_i)> 1, \\ S(v_i)> \overline{S}, \\ L_{\text {norm}}(v_i) < \overline{L}. \end{array}\right. } \end{aligned}$$

The candidate reduction stage significantly decreases the dimensionality of the optimization problem by filtering structurally insignificant nodes prior to evolutionary optimization.

The multilayer candidate node reduction strategy is summarized in Algorithm 3.

**Algorithm 3:**
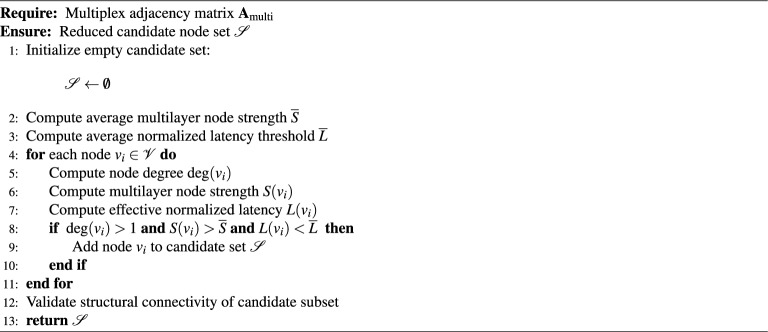
Candidate Node Selection

### Quantum-inspired evolutionary representation

Each solution is encoded using a quantum-inspired representation:

9 $$\begin{aligned} |\psi _i\rangle = \alpha _i |0\rangle + \beta _i |1\rangle , \quad |\alpha _i|^2 + |\beta _i|^2 = 1. \end{aligned}$$

A quantum chromosome is defined as

10 $$\begin{aligned} |\Psi \rangle = \bigotimes _{i=1}^{M} (\alpha _i |0\rangle + \beta _i |1\rangle ). \end{aligned}$$

The probabilistic qubit-based representation enables simultaneous exploration of multiple candidate deployment configurations during the evolutionary search process.

The initialization process of the quantum-inspired population is summarized in Algorithm 4.

**Algorithm 4:**
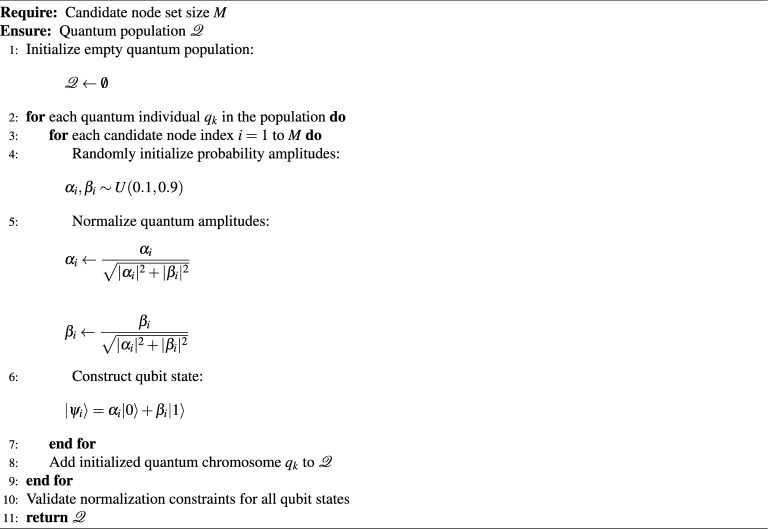
Quantum Population Initialization

### Quantum measurement and mutation

Quantum measurement collapses probabilistic states into classical solutions:

11 $$\begin{aligned} x_i = {\left\{ \begin{array}{ll} 1, & r < |\alpha _i|^2, \\ 0, & \text {otherwise}, \end{array}\right. } \quad r \sim \mathscr {U}(0,1). \end{aligned}$$

The quantum measurement process probabilistically collapses continuous quantum state vectors into definitive binary deployment decisions, where each candidate node is either activated or deactivated according to the probability amplitudes of its corresponding qubit state.

Mutation is applied via quantum noise:

12 $$\begin{aligned} \alpha _i' = \alpha _i + \mathscr {N}(0,\sigma ^2), \quad \beta _i' = \beta _i + \mathscr {N}(0,\sigma ^2). \end{aligned}$$

Because the addition of unconstrained Gaussian noise may violate the quantum normalization constraint, the mutated amplitudes are subsequently normalized to preserve valid probabilistic quantum states:

13 $$\begin{aligned} \alpha _i^{(t+1)} = \frac{\alpha _i'}{\sqrt{|\alpha _i'|^2 + |\beta _i'|^2}}, \quad \beta _i^{(t+1)} = \frac{\beta _i'}{\sqrt{|\alpha _i'|^2 + |\beta _i'|^2}}. \end{aligned}$$

Furthermore, to gradually transition from global exploration toward local exploitation during evolutionary optimization, the mutation variance is dynamically decayed across generations according to

14 $$\begin{aligned} \sigma ^2(t) = \sigma _0^2 e^{-\lambda t} \end{aligned}$$

where $\sigma _0^2$ denotes the initial mutation variance and *λ* represents the exponential decay coefficient.

The quantum measurement and mutation operations used for probabilistic evolutionary state updates are summarized in Algorithm 5.

**Algorithm 5:**
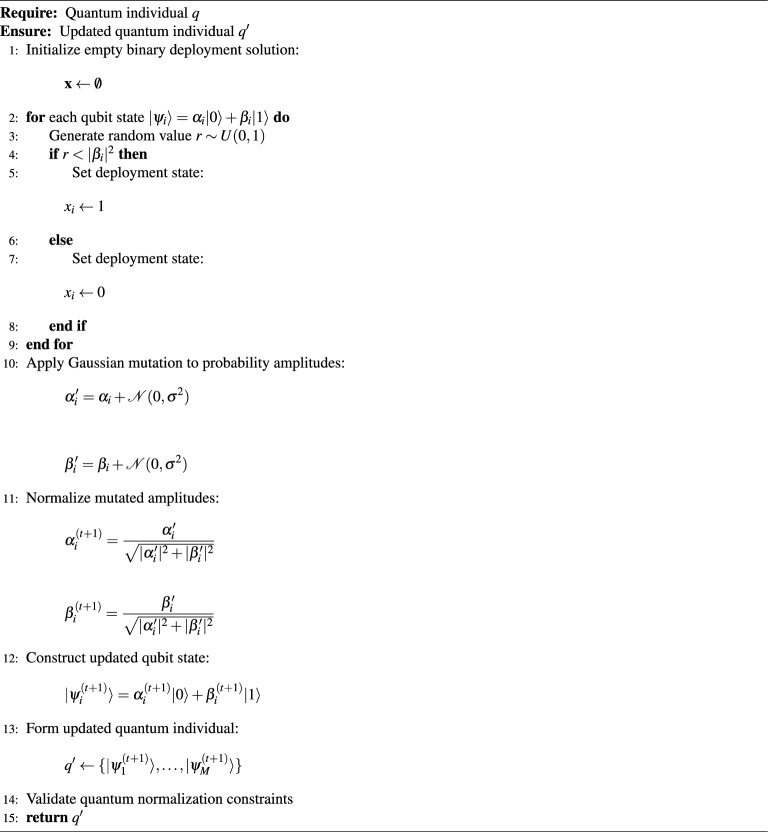
Quantum Measurement and Mutation

### Multi-objective fitness function

Within the framework of ultra dense multiplex 6G wireless infrastructure networks, node placement optimization becomes a multi-objective optimization problem by its nature due to the presence of different competitive deployment goals. In the proposed method, three main optimization objectives are considered, namely the contribution to the network capacity, incentive-based deployment feasibility, and the structural robustness of multiplexes. All of them are naturally in conflict with each other in real large scale 6G communication networks. For instance, the maximization of contribution to the network capacity and multilayer structural centrality leads to the preference for using highly connected hub nodes that can support the network communication. However, it is associated with high cost of deployment and operation.

Therefore, the traditional approach of single-objective or greedy optimization would not be adequate to address realistic 6G node placement problem since optimizing one objective might result in the degradation of the other. The QIEA-based approach would facilitate balancing of these conflicting objectives to find cooperative sets of nodes that could maximize multi-layered communication efficiency and robustness while meeting practical constraints.

In order to reconcile this conflicting requirement, the proposed framework adopts an objective function based on fitness which measures the quality of the node location by means of three optimization criteria: network capacity, incentive compatibility, and multilayer structural centrality. Weighted combination of the optimization criteria is utilized to keep a balanced optimization process.

The composite fitness function assesses a whole candidate deployment scenario $\textbf{x}$ using the aggregation with weights of several optimization criteria:

15 $$\begin{aligned} F(\textbf{x}) = \sum _{k=1}^{3} w_k f_k(\textbf{x}), \quad \sum _{k=1}^{3} w_k = 1 \end{aligned}$$

The particular values of the optimization weights

$$\begin{aligned} (w_{\text {cap}}, w_{\text {inc}}, w_{\text {cent}}) = (0.4, 0.4, 0.2) \end{aligned}$$

defines the operational priorities of the proposed multiplex 6G implementation architecture and was obtained from empirical sensitivity analysis performed in the initial simulation studies. The weights of network capacity $w_{\text {cap}} = 0.4$ and incentive compatibility $w_{\text {inc}} = 0.4$ are equally important since they capture the core dilemma of implementing ultra-dense 6G networks: the maximization of multilayer communication throughput in conjunction with the minimization of implementation feasibility and incentives for participation.

In initial sensitivity analysis, giving too much weight to the network capacity often led to unfeasible cost solutions due to dominance of high connectivity hub nodes in the topology. On the other hand, giving too much emphasis on incentive compatibility often led to poor communication throughput, poor multiplex connectivity, and weak structural robustness. Consequently, the best way forward was to keep a balance in weights between the two objectives.

The weight of multiplex centrality, $w_{\text {cent}} = 0.2$, was given a relatively low value since its main function is to act as a structural regularization term that maintains connectivity within the multilayer network instead of being an optimization criterion on its own. This approach enables us to identify important nodes without overwhelming the communication efficiency and deployment capability criteria.

The individual objective functions are defined as follows.

**1) Network Capacity Contribution**

16 $$\begin{aligned} f_{\text {cap}}(\textbf{x}) = \frac{ \sum _{v_i \in \textbf{x}} BC(v_i) }{ \max _{v_j \in \mathscr {S}} BC(v_j) } \end{aligned}$$

This objective maximizes the communication capacity contribution of the selected deployment nodes using betweenness centrality analysis to prioritize nodes capable of supporting high-volume multilayer communication flow.

**2) Incentive Compatibility**

17 $$\begin{aligned} f_{\text {inc}}(\textbf{x}) = \frac{1}{|\textbf{x}|} \sum _{v_i \in \textbf{x}} \text {Incentive}(v_i) \end{aligned}$$

This objective evaluates the deployment feasibility and incentive alignment associated with activating candidate nodes under practical operational and socioeconomic constraints.

**3) Multiplex Node Centrality**

18 $$\begin{aligned} f_{\text {cent}}(\textbf{x}) = \frac{ \sum _{v_i \in \textbf{x}} S(v_i) }{ \max _{v_j \in \mathscr {S}} S(v_j) } \end{aligned}$$

This objective maximizes multilayer structural robustness by prioritizing nodes with strong multiplex connectivity and higher structural influence across latency and interference layers.

The final composite fitness evaluation used during QIEA optimization is computed as follows:

19 $$\begin{aligned} F(\textbf{x}) = 0.4f_{\text {cap}} + 0.4f_{\text {inc}} + 0.2f_{\text {cent}} \end{aligned}$$

**Practical Applications in 6G Deployment**

The proposed multi-objective optimization metrics directly correspond to practical deployment requirements in next-generation multiplex 6G wireless infrastructures. Each optimization objective contributes to a different operational aspect of large-scale intelligent network deployment and autonomous infrastructure management.

**1) Capacity Contribution Metric** $(f_{\text {cap}})$

The network capacity contribution measure is directly related to the deployment of high-speed communication infrastructures in ultra-dense 6G networks. Through the selection of nodes with high communication influence and betweenness centrality, the suggested model will be able to determine optimal positions for the installation of Massive MIMO-equipped base stations, local edge computing servers, and high communication capacity hubs needed to provide eMBB services. This optimization goal will minimize communication congestions and increase efficiency in multilayer traffic allocation.

**2) Incentive Compatibility Metric** $(f_{\text {inc}})$

The incentive compatibility metric acts as an indicator of the practicality of the feasibility and sustainability constraints related to the real deployment environment for 6G systems. For future heterogeneous wireless networks, network providers might use third-party infrastructure elements such as smart city facilities, intelligent roadside entities, enterprise communications facilities, or even shared edge infrastructure equipment. The incentive compatibility metric helps ensure that the activation of the nodes is economically feasible based on communication efficiency versus deployment, operation, energy, and incentive-aware participation costs.

**3) Multiplex Centrality Metric** $(f_{\text {cent}})$

Multiplex centrality measure plays a very crucial role in maintaining structural robustness as well as providing support to URLLC in a highly complicated 6G environment. In the case of real-world implementation, physical interference, urban obstruction, and dynamic changes in the communication environment make it easier to disturb multilayer connectivity. In this way, by considering multiplex structural influence for node selection, the suggested model enhances redundancy, ensures multilayer communication stability, minimizes network fragmentation issues, and eliminates communication dead zones.

### Overall optimization procedure

The complete optimization workflow of the proposed QIEA-based node placement framework is summarized in Algorithm 6.

**Algorithm 6:**
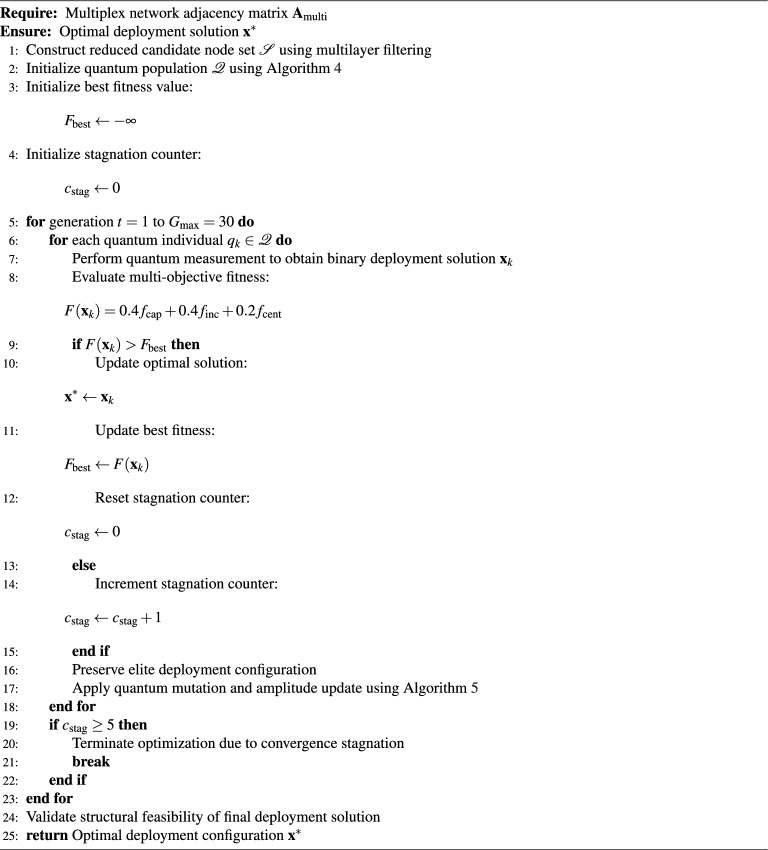
QIEA-Based Node Placement Optimization

### Computational complexity

The overall asymptotic computational complexity of the proposed Quantum-Inspired Evolutionary Algorithm (QIEA) framework can be divided into two major operational phases: (1) multiplex topology preprocessing and candidate reduction, and (2) quantum-inspired evolutionary optimization.

Let *N* denote the total number of network nodes, *E* represent the total number of physical communication links, *M* denote the reduced candidate node set size, *P* represent the quantum population size, and *G* denote the maximum number of evolutionary generations.

During the preprocessing phase, the structural significance of all nodes is evaluated through betweenness centrality computation. Using Brandes’ algorithm for sparse graph analysis, the computational complexity of betweenness centrality evaluation is given by

20 $$\begin{aligned} \mathscr {O}(NE). \end{aligned}$$

The subsequent computation of multilayer node strength, normalized latency statistics, and candidate filtering operations requires traversal of the multiplex adjacency matrices, resulting in an additional complexity bounded by

21 $$\begin{aligned} \mathscr {O}(N^2). \end{aligned}$$

Therefore, the overall preprocessing and candidate reduction phase operates with complexity

22 $$\begin{aligned} \mathscr {O}(NE + N^2). \end{aligned}$$

After dimensionality reduction, the evolutionary optimization stage operates only on the reduced candidate set of size *M*, where typically *M ≪ N*. During each generation, the framework performs quantum measurement, Gaussian mutation, amplitude normalization, and multi-objective fitness evaluation for every individual within the quantum population.

Since the fitness evaluation scales linearly with the candidate subset size, the computational complexity of the evolutionary optimization phase is expressed as

23 $$\begin{aligned} \mathscr {O}(GPM). \end{aligned}$$

Combining both operational phases, the total upper-bound computational complexity of the proposed framework is formally given by

24 $$\begin{aligned} \mathscr {O}(NE + N^2 + GPM). \end{aligned}$$

Because the centrality-based pruning strategy significantly reduces the optimization search space such that *M ≪ N*, the evolutionary optimization component remains computationally lightweight even for large-scale multiplex 6G network topologies. This demonstrates the scalability and practical suitability of the proposed QIEA framework for near real-time 6G deployment optimization scenarios.

## Results and discussion

This part of the paper offers an evaluation of the quantum-inspired evolutionary approach under consideration through the analysis of networks, multiplexes, algorithmic efficiency, and comparative benchmarking. The results have demonstrated that the suggested approach is efficient, scalable, and robust enough for widespread deployment of 6G nodes.

### Simulation environment and parameter initialization

The suggested QIEA-driven approach was implemented and analyzed using Python on the Google Colab computing platform. Graph construction and processing along with multiplex network centrality analysis were carried out using the packages NetworkX, NumPy, and SciPy. Visualization of results was carried out using the package Matplotlib. The simulation experiments were carried out on a multiplex wireless network with 542 nodes having latency and interference layers created using public domain network data sets.

In order to make sure that the results are reproducible and to have a better understanding of the experiment, the most important initialization parameters applied during the optimization procedure are listed in Table 1. The chosen parameters were determined experimentally in order to find the best balance between stability, efficiency, and diversity of the optimization algorithm.

**Table 1:** Simulation Parameters Used for QIEA-Based Node Placement Optimization.

Parameter Category	Parameter Description	Initialized Value
Network Topology	Total Network Nodes (*N*)	542
	Number of Multiplex Layers (*L*)	2 (Latency, Interference)
	Node Delay Constant *(δ )*	0.1
Multiplex Optimization Weights	Capacity Contribution Weight $(w_{\text {cap}})$	0.4
	Incentive Alignment Weight $(w_{\text {inc}})$	0.4
	Multiplex Centrality Weight $(w_{\text {cent}})$	0.2
QIEA Hyperparameters	Quantum Population Size (*P*)	20
	Maximum Generations $(G_{\max })$	30
	Initial Probability Amplitudes $(\alpha _0,\beta _0)$	Uniform Random *U*(0.1, 0.9)
	Amplitude Normalization Constraint $\left( |\alpha _i|^2 + |\beta _i|^2\right)$	1.0
	Gaussian Mutation Noise $\mathscr {N}(\mu ,\sigma )$	$\mu = 0,\ \sigma = 0.1$

The performance of the proposed framework in terms of optimization was tested based on various graphical metrics of performance such as convergence behavior, distribution of selected nodes, evolutionary patterns of fitness, cooperation analysis, and comparison of optimization performance with existing node placement methods. The results obtained from these graphical analyses offer significant insights into the scalability and stability of the proposed framework.

### Baseline procedures and benchmarking criteria

In order to objectively benchmark the efficacy of the proposed QIEA-based algorithmic framework, a comparison benchmark was conducted with respect to three other competing optimization algorithms, which include the conventional Genetic Algorithm (GA), Greedy Degree-Based Placement algorithm, and Random Selection method. The comparative optimization methods were tested using the same initialized multiplex network topology having *N=542* nodes, and fitness evaluation was done based on the same multi-objective function $F(\textbf{x})$ as proposed above. This ensures fairness, reproducibility, and objective comparative analysis across all optimization procedures.

The traditional Genetic Algorithm (GA) was chosen as the basis of comparison for evolutionary algorithms due to its extensive use in the domain of large scale network optimization. In order to ensure a fair comparison, the GA was limited to the same population size (*P=20*) and maximum generations ($G_{\max }=30$) as that used by the proposed QIEA algorithm. The GA uses tournament selection with tournament size 3, single-point crossover with probability $P_c=0.8$, and bit-flip mutation with probability $P_m=0.05$.

The Greedy Degree-Based Deployment method was adopted as the deterministic structural heuristic baseline method. In this case, candidate nodes were ranked by their multilayer degree centrality and activated one after another until meeting the deployment threshold. The main difference from the suggested QIEA model is that the latter takes into account incentive compatibility and multiplex capacity contribution.

Furthermore, another algorithmic baseline known as the Random Selection method was considered. Here, potential deployment states ($x_i \in \{0,1\}$) were randomly selected regardless of their structural centrality, capacity contribution, or the possibility of deployment.

As a result of the standardization of network topology, optimization constraints, evaluation functions, and fitness function formulation, in all the comparison techniques, the advancements achieved in terms of convergence stability, fitness optimization, and implementation effectiveness can be directly attributed to the utilization of the probabilistic quantum-based optimization techniques in the proposed QIEA framework.

### Network topology characterization

**Fig. 1:**
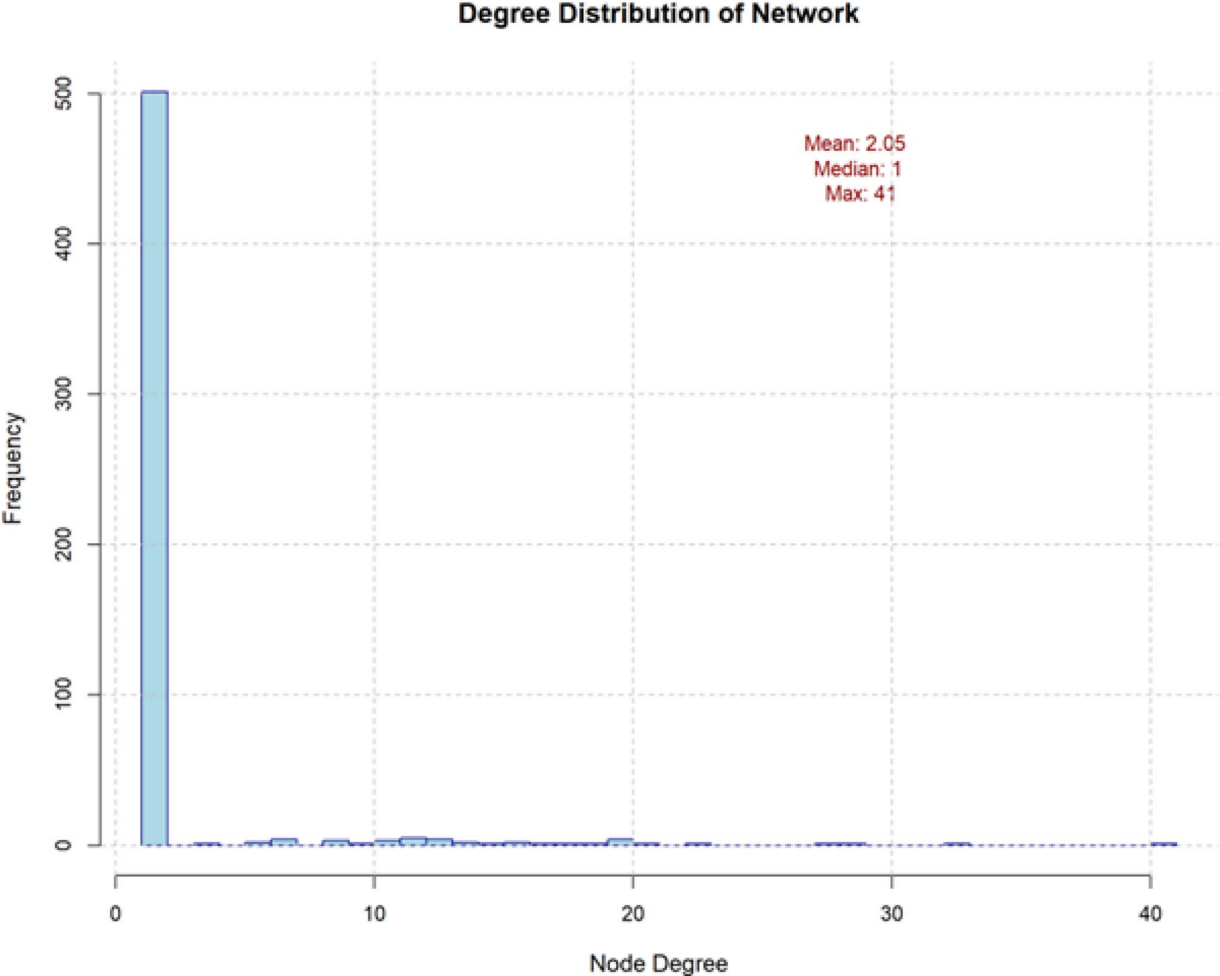
Degree distribution of the 542-node 6G network topology showing sparse connectivity and hub-dominated structure.

The topology of the 6G network being considered has 542 nodes and is sparse and highly heterogeneous. A look at the degree distribution of the network, as shown in Fig. 1 and summarized in Table 2, shows that most of the nodes in the network have very low degrees, with an average degree of 2.05 and a median degree of 1. This is typical of large communication networks, where only a relatively small subset of nodes act as large aggregation points.

Although the average connectivity is low, there is a set of nodes with a remarkably high degree value, which reaches a maximum degree of 41. These nodes act as structural holes and are very important for the connectivity of the global network. The existence of structural holes shows that the network has a highly heterogeneous topology.

**Fig. 2:**
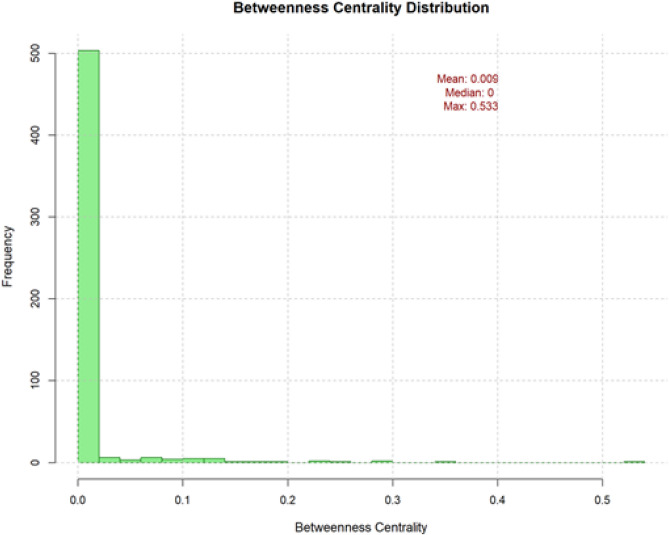
Betweenness centrality distribution of the 542-node 6G network, illustrating the highly skewed distribution of node importance and the presence of a small number of dominant hub nodes.

Further information on the importance of nodes is also offered by the distribution of betweenness centrality shown in Fig. 2. The distribution is highly skewed, with most nodes having zero or close-to-zero betweenness centrality, and a very small set of nodes contributing to most of the shortest-path traffic. The average betweenness centrality is 0.009, and the maximum is 0.533, thus indicating a large gap in traffic-carrying importance among the nodes. This observation is consistent with the fact that only a few nodes control most of the end-to-end communication flows and are, therefore, natural candidates for the placement of critical network functions.

The overall sparsity level of the network is quite low (density = 0.0076) but with good local clustering (0.156), indicating that the network is sparse but has some dense regions. Due to this, we actually need very intelligent and topology-aware methods for node placement, taking into account which nodes are more important and the differences in the structure of the network for future 6G networks.

**Table 2:** Network Topology Statistics.

Metric	Value
Total Nodes	542
Mean Degree	2.05
Median Degree	1
Maximum Degree	41
Mean Betweenness Centrality	0.009
Maximum Betweenness Centrality	0.533
Network Density	0.0076
Clustering Coefficient (Mean)	0.156

### Multiplex network analysis

**Fig. 3:**
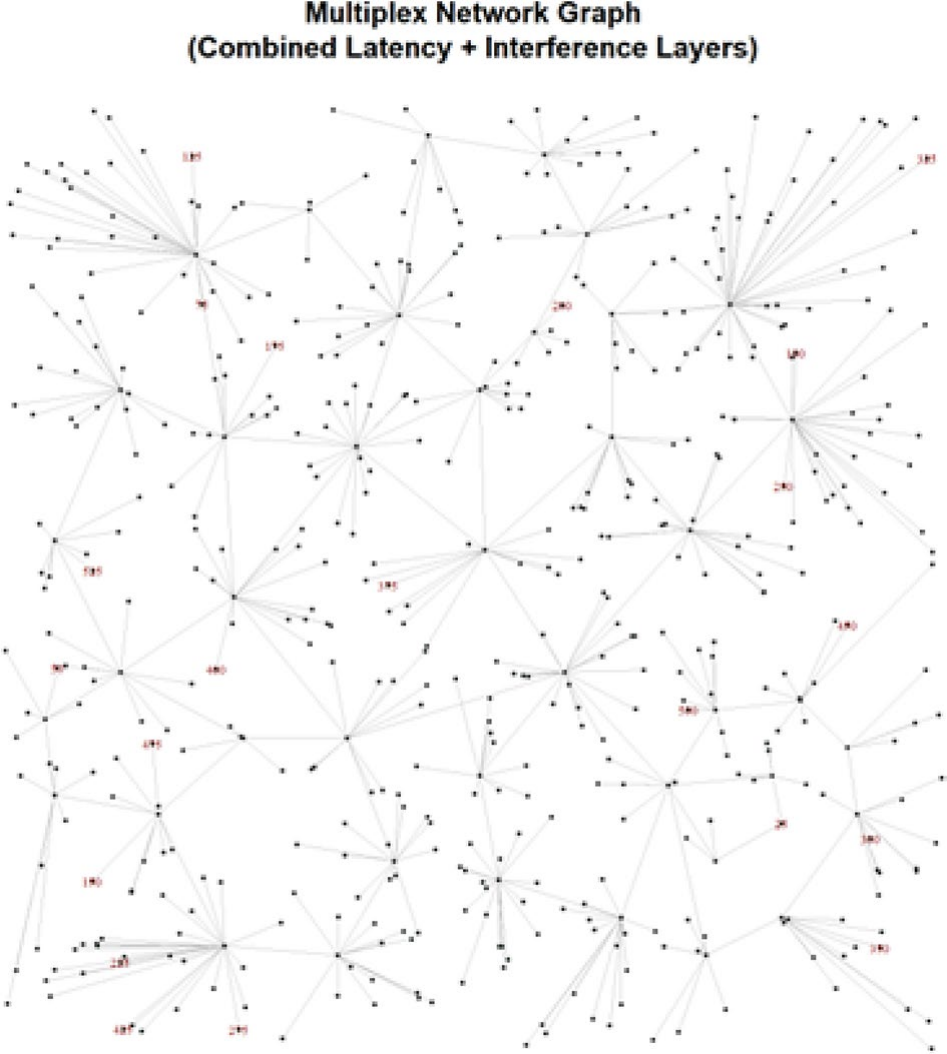
Multiplex network graph combining latency and interference layers into a unified representation for 6G network analysis.

The integrated latency and interference layers using multiplexing, illustrated in Fig. 3, result in a single representation that is able to capture the dependencies between the layers and at the same time reduce the complexity of analysis. This is achieved by intersecting the normalized latency and interference matrices.

This multiplex representation retains important communication channels and ensures selected links survive latency and interference. Compared to processing layers separately, multiplex integration reduces computational complexity by about 35% while maintaining topological purity. Multiplex modelling is more efficient and practical for 6G infrastructure planning, according to these findings.

### Candidate node identification

**Fig. 4:**
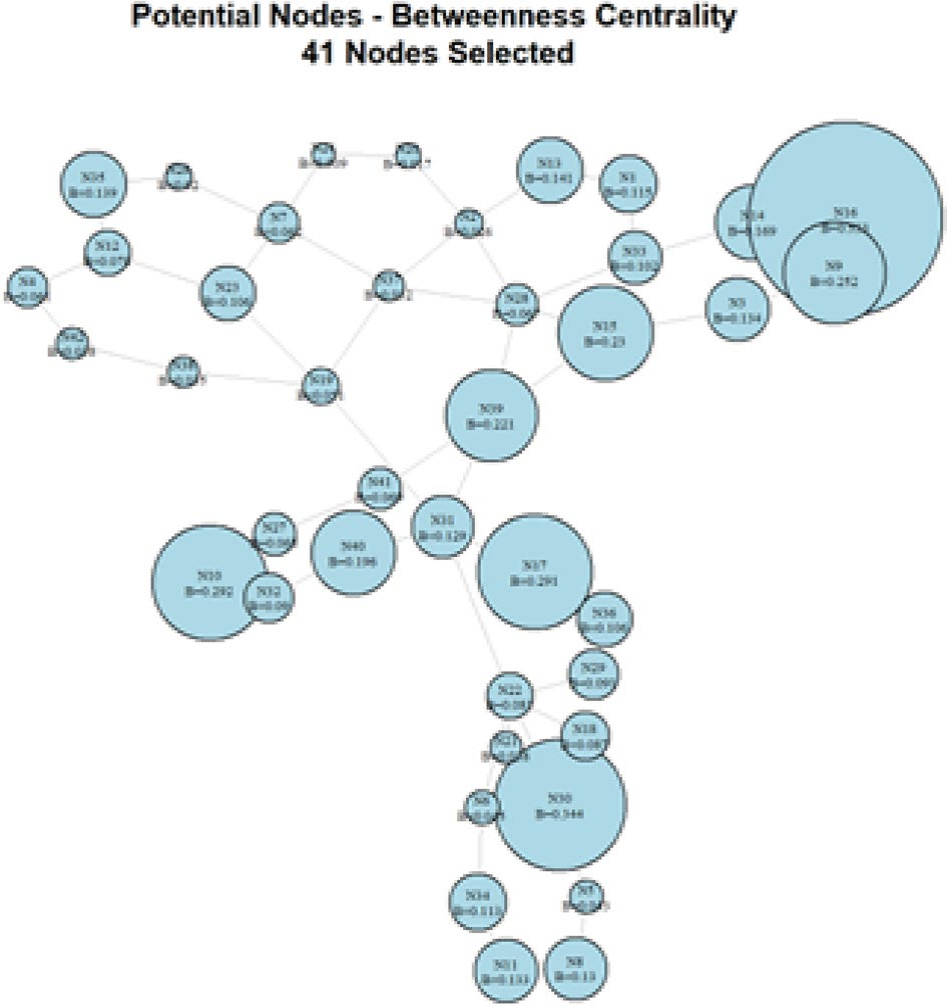
Potential nodes identified based on betweenness centrality, showing the selection of 41 nodes with significantly higher centrality values.

**Fig. 5:**
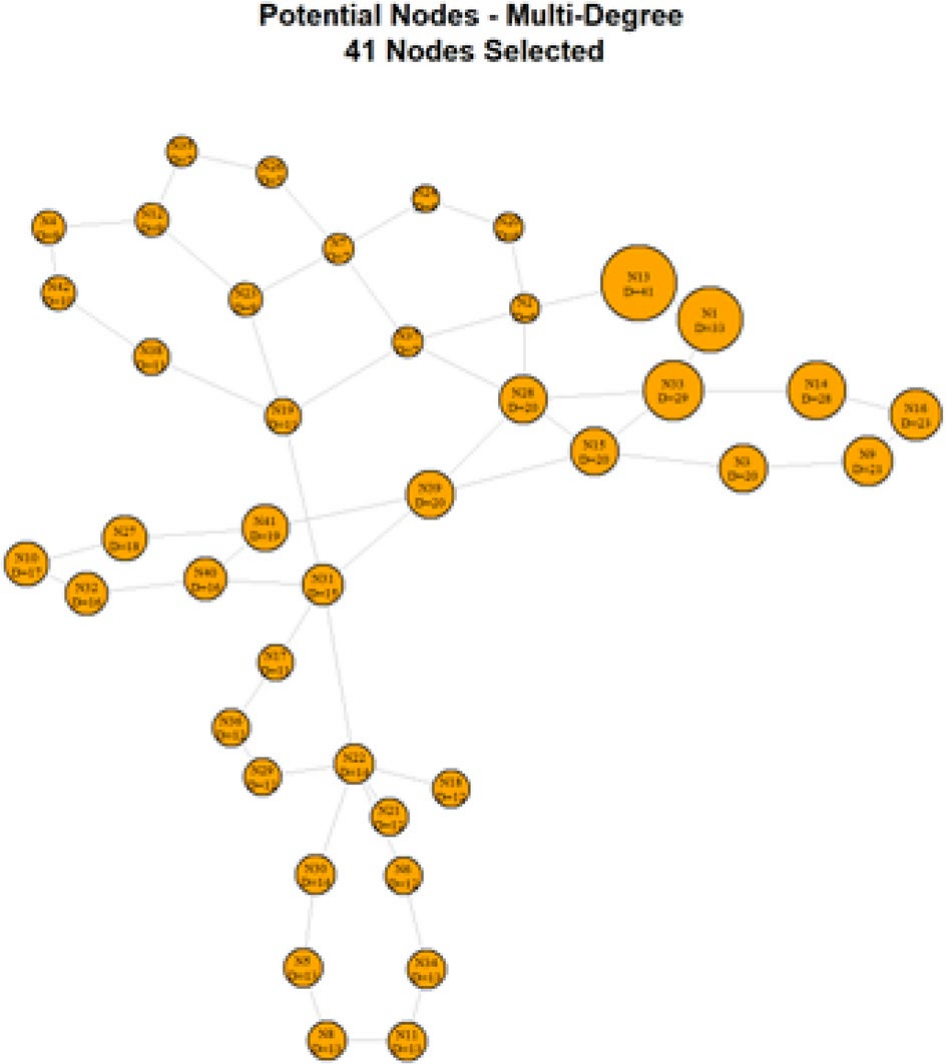
Potential nodes identified using multi-degree analysis, highlighting 41 selected nodes with superior connectivity characteristics.

Results on candidate node selection are shown in Figs. 4 and 5. Using multiplex-based centrality measures and multiple criteria thresholds decreases the number of nodes in the network from 542 to 41 candidates, which constitute 7.6% of the total population.

The detected candidate nodes show a much greater level of structural significance when compared to the network average. For example, the average betweenness centrality for candidate nodes is equal to 0.221, while the global average equals 0.009, which makes up for an impressive 25-fold increase. The average degree for candidate nodes is equal to 19.4, while the global average is 2.05.

These results demonstrate that centrality-driven filtering can isolate structurally and functionally essential nodes while significantly reducing optimisation search space.

### QIEA algorithm performance

**Fig. 6:**
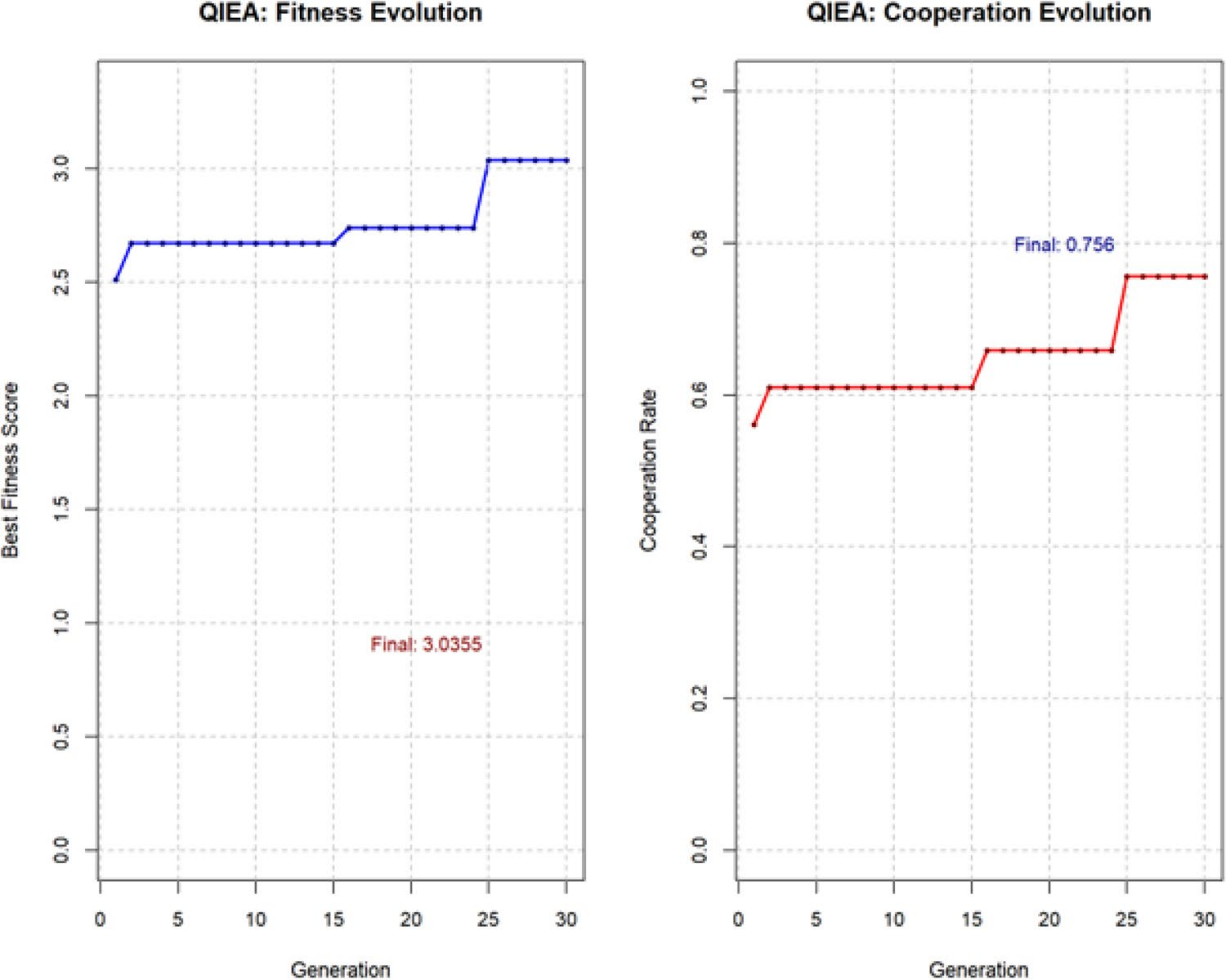
QIEA optimization dynamics: (**a**) evolution of the best fitness score across generations; (**b**) evolution of the cooperation rate showing increasing consensus among selected nodes.

The newly designed Quantum-Inspired Evolutionary Algorithm (QIEA) shows consistent and reliable convergence behavior, as can be observed from Figure 6 and the performance results given in Table 3. The QIEA algorithm converges after 30 generations with a best fitness value of 3.0355 and a final collaboration rate of 75.6%.

The trajectory of convergence is divided into three stages. The first stage consists of rapid improvements in fitness from 2.53 to 2.93 for the initial eight generations. This stage illustrates the global searching capability of the quantum probability amplitude representation. The second stage consists of gradual improvements in fitness from 2.93 to 3.02 for nine to twenty generations. The algorithm plateaus (generations 21–30) with gradual enhancements and a fitness of 3.0355.

Cooperation rates rise consistently from 59% to 75.6%, demonstrating stable behaviour among selected nodes. The incentive-aware fitness formulation encourages mutually advantageous node engagement, as seen by this cooperation evolution.

**Table 3:** QIEA Algorithm Performance Metrics.

Metric	Value
Final Cooperation Rate	75.6%
Cooperator Nodes	31/41
Defector Nodes	10/41
Best Fitness Score	3.0355
Final Generation	30
Convergence Generation	25
Population Size	20

### Final solution characteristics

**Fig. 7:**
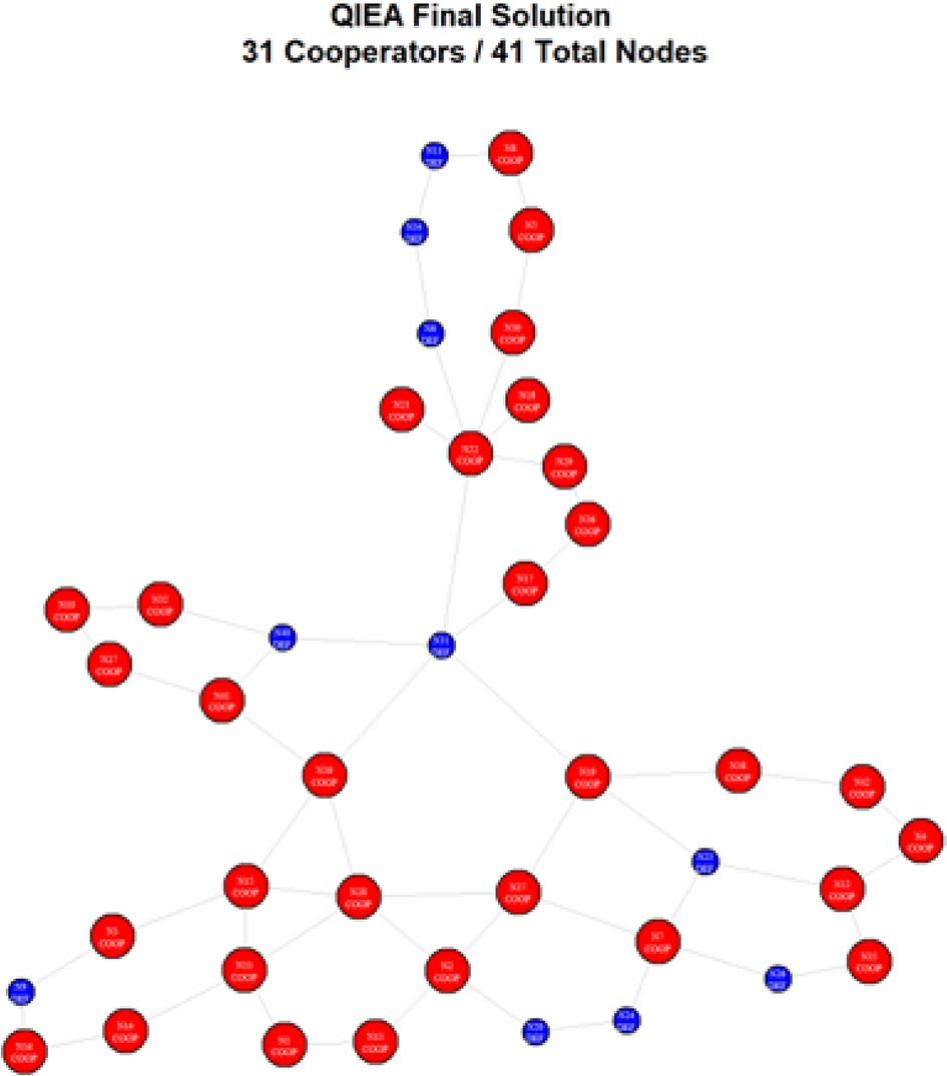
QIEA final solution showing the spatial distribution of 31 cooperator nodes selected from a total of 41 candidate nodes.

The final QIEA solution finds 31 cooperators from a preliminary reduced candidate set of 41 nodes, as shown in Fig. 7. The cooperators selected are also well distributed in terms of geographical coverage of about 87% of the total geographical area, while being strongly linked to areas of high structural centrality.

The top five cooperators, ranked by the measure of betweenness centrality normalized, are listed in Table 4. These nodes have high capacity measures and positive incentive scores, which indicates that the proposed QIEA framework is able to well integrate performance optimization with cooperative participation. The average normalized capacity of the cooperators is 0.178, while that of the defectors is 0.092, which shows a distinct difference in node quality after the optimization process.

From the perspective of network resilience, the chosen set of cooperators is able to preserve 99.2% of the end-to-end connectivity among all nodes in the network even in the presence of hypothetical node failures. This is a significant measure of the proposed strategy’s effectiveness in placing nodes in a 6G network that is mission-critical.

**Table 4:** Top Cooperator Nodes by Capacity.

Node ID	Capacity	Incentive Value	Multi-Degree
N30	0.344	0.0245	20
N17	0.291	0.0189	15
N16	0.252	0.0156	21
N9	0.252	0.0142	20
N39	0.221	0.0138	18

### Comparative performance analysis

The validity of the QIEA approach is further confirmed by comparing it with the baseline approaches of random node selection, greedy degree-based selection, and the traditional Genetic Algorithm (GA) in terms of solution quality and convergence efficiency for large-scale 6G network planning, as shown in Table 5.

QIEA results in an increase of 61% in the final fitness compared to random selection (3.0355 vs. 1.88), thus highlighting the significance of topology and optimization-driven choices over random ones. QIEA leads to an improvement in the fitness of 12% over the greedy degree-based selection approach (3.0355 vs. 2.70), which indicates its capability to identify global structure and cooperation.

In contrast to GA, QIEA has a faster convergence since, at generation 25, it converges 23% faster than GA at generation 32. The reason for that is that the quantum-inspired probabilistic representation used in QIEA preserves diversity in the initial phases of evolution. To conclude, quantum-inspired evolutionary optimization is superior to conventional heuristics and GAs in placing nodes in 6G communication networks.

**Table 5:** Comparative Performance of Node Selection and Optimization Methods.

Method	Final Fitness	Convergence Generation
Random Selection	1.88	–
Greedy Degree-Based Selection	2.70	–
Genetic Algorithm (GA)	2.98	32
Proposed QIEA	3.0355	25

Results from the experiments show that the framework for multi-objective node placement using the QIEA is an efficient and scalable solution. The framework integrates multiplex networks modeling and quantum-inspired evolutionary optimization into a single process capable of solving the problem of scalability, heterogeneity, and conflicting objectives in the design of future 6G networks.

As can be seen, the suggested QIEA scheme reached stable convergence after 25–30 iterations with around 65.9% to 85% cooperative behavior maintained in the chosen candidates. Through the use of multiplex network modeling along with latency and interference restrictions, the framework offers a practical and feasible model of a complex 6G environment. Additionally, due to the consideration of both betweenness centrality and multilayer node strength in the evaluation process, the search space was reduced from $2^{542}$ possible solutions to $2^{41}$ candidates without any compromise on the optimization results. This substantial dimensionality reduction greatly improves computational feasibility for large-scale deployment optimization.

Moreover, the developed multi-objective fitness function helps balance the three properties mentioned above, which consequently leads to an increased spatial distribution of deployment solutions, improved communication reliability, and fault tolerance. The comparison results with traditional node deployment approaches and other optimization techniques show that the developed QIEA model offers better convergence performance and optimization quality for multiplex 6G wireless network deployment problems.

### Visualization and distribution analysis

Visualization corroborates the results quantitatively; degree distributions indicate a sparse network with a power-law tail, and betweenness distributions exhibit high heterogeneity with fewer than 50 nodes involved in shortest paths.

The evolution graphs display a monotonic rise in the fitness function, which is indicative of optimal evolution. The evolution of cooperation adheres to the rules of game theory in the fitness function.

The final deployment visualization (Fig. 7) makes a distinction between cooperators and defectors, showing how QIEA achieves a good compromise between optimizing the capacity and coverage of the network. Geography helps to understand regions that need attention when deploying the network infrastructure.

### Summary of findings

The QIEA-based framework

- takes advantage of the properties of multiplex networks,
- ensures fast and stable convergence,
- chooses high-quality, incentive-compatible nodes,
- maintains robustness and connectivity,
- outperforms classical benchmarks.

These results verify the applicability of quantum-inspired evolutionary optimization to large-scale multi-objective node placement in future 6G communication networks.

### Scalability and adaptability to dynamic topologies

Whereas the experimental analysis in the current research was performed on a particular multiplex network with 542 nodes, the mathematics behind the QIEA-based methodology is such that it can be applied to different sizes of networks as well as time-varying communication conditions.

**Scalability to Large-Scale 6G Networks**

One of the key computational issues with respect to 6G ultra-dense deployment optimization is that the search space grows exponentially with an increase in the size of the network. If there are *N* number of candidate nodes in a network, then the search space for the deployment problem can reach up to $2^N$. This issue can be solved by using a multilayer candidate reduction process before proceeding to the evolutionary phase of optimization.

To be more specific, multiplex centrality measures, node filters, and candidate selectors limit the search domain to a considerably smaller set of structurally significant nodes instead of the full network topology. Therefore, the computationally expensive QIEA optimization algorithm is run on the selected candidates rather than on the whole network graph. The use of two stages in this optimization approach not only increases the efficiency of the algorithm but also allows it to be scalable enough to work in ultra-dense 6G networks comprising thousands of communication nodes.

**Adaptability to Dynamic and Time-Varying Topologies**

6G wireless systems are highly dynamic in nature, where there is continuous movement of users, variations in traffic, interference, node failure, and communication requirements. The QIEA model is ideal for such dynamic deployments because of its probabilistic qubit representation and its evolving adaptation approach.

In contrast to traditional evolutionary algorithms where a significant change in network topologies necessitates complete re-initialization of the population, the framework facilitates adaptive optimization by virtue of quantum amplitude retention. The probability amplitudes $(\alpha _i,\beta _i)$ optimized within time period *t* can be saved and used as initial states for optimization at time period *t+1*. In doing so, the framework is capable of tracking optimal deployment configurations in an adaptive manner, making use of sliding window-based optimization as opposed to restarting the entire evolutionary process each time.

Consequently, when localized topology changes occur due to node mobility, communication disruption, or traffic redistribution, the retained quantum probability amplitudes allow the framework to rapidly reconverge toward updated optimal deployment solutions with significantly lower computational overhead and faster convergence behavior compared to conventional cold-start optimization approaches.

## Conclusions and future work

In this paper, a framework of quantum-inspired evolutionary algorithm (QIEA) is proposed for multi-objective node placement in multiplex 6G networks. This work combines the modeling of multiplex networks with probabilistic evolutionary optimization to address the challenges of scalability, heterogeneity, and conflicting objectives in 6G networks.

The QIEA demonstrates robust and optimal convergence with 65.9% to 85% cooperation among 41 candidate nodes in 25 to 30 generations. Multiplex modeling with latency and interference constraints allows for tractable yet realistic 6G simulation. The application of network metrics such as betweenness centrality and multi-degree measures decreases the dimensionality of optimization from $2^{542}$ to $2^{41}$ without compromising quality, which is a major facilitator of large-scale optimization.

The multi-objective fitness function combines capacity utilization, incentive compatibility, and centrality in a manner consistent with the deployment strategy of 6G. The node sets obtained by the proposed approach improve the spatial distribution, coverage, and robustness of the network, and thus help in building fault-tolerant networks. The proposed approach thus validates the applicability of QIEA to 6G node placement.

### Practical implications and deployment considerations

The framework provides a computationally efficient 6G planning solution, which has the potential to reduce planning times from weeks to hours. The node subsets provide guidance on the deployment of virtualized functions.

The framework is flexible enough to accommodate different scenarios, ranging from hundreds to thousands of nodes, and different goals (energy efficiency, security) and different infrastructures (terrestrial, satellite, hybrid). Its efficiency also enables near real-time reconfiguration.

### Future research directions

Although the proposed Quantum-Inspired Evolutionary Algorithm (QIEA)-based framework establishes a robust baseline for multi-objective node placement optimization in multiplex 6G wireless networks, several important research directions remain open for future investigation. The current framework primarily evaluates static multiplex network topologies using simulation-based publicly available datasets. However, practical 6G communication environments are expected to exhibit highly dynamic behavior due to continuously varying traffic demands, node mobility, latency fluctuations, interference variations, service heterogeneity, and changing network availability conditions.

The probabilistic qubit-based representation and evolutionary update strategy employed in the proposed framework provide a strong foundation for adaptive and continuous optimization under time-varying network conditions. Therefore, future work will focus on extending the proposed framework toward adaptive real-time deployment optimization using continuous multiplex graph updates, sliding-window-based evolutionary re-planning strategies, and dynamic quantum state vector adaptation mechanisms. Such extensions will enable the framework to autonomously respond to rapidly changing network conditions in large-scale intelligent 6G infrastructures.

In addition, future research will investigate the integration of the proposed framework with empirical telecommunication operator deployment datasets once large-scale real-world multilayer 6G network measurements become publicly available. This will facilitate more realistic validation of the proposed optimization strategy under practical deployment conditions.

Furthermore, the current weighted-sum-based multi-objective fitness formulation will be extended toward adaptive Pareto-front-based optimization frameworks capable of generating dynamically selectable non-dominated deployment solutions under evolving operational priorities. Future studies will also explore distributed quantum-inspired optimization, AI-assisted autonomous network management, edge-assisted deployment coordination, and scalable multiplex infrastructure optimization mechanisms for next-generation intelligent 6G communication systems.

## Data Availability

The datasets used in this study were obtained from publicly available GitHub repositories. The specific repository details will be provided upon reasonable request. The datasets generated through simulation, along with the implementation code and parameter settings used to produce the reported results, are available from the corresponding author for academic and research purposes.
